# Opportunities for Single-Cell Sequencing Technologies and Data Science

**DOI:** 10.3390/cancers12113433

**Published:** 2020-11-19

**Authors:** Lisa Maria Mustachio, Jason Roszik

**Affiliations:** 1 Department of Epigenetics and Molecular Carcinogenesis, The University of Texas MD Anderson Cancer Center, Houston, TX 77030, USA; 2 Center for Cancer Epigenetics, The University of Texas MD Anderson Cancer Center, Houston, TX 77030, USA; 3 Department of Genomic Medicine, Division of Cancer Medicine, The University of Texas MD Anderson Cancer Center, Houston, TX 77030, USA; 4 Department of Melanoma Medical Oncology, Division of Cancer Medicine, The University of Texas MD Anderson Cancer Center, Houston, TX 77030, USA

This Special Issue on “Single-cell Data Science” aims to highlight recent advances in the area of single-cell sequencing technologies and data analytics. Single-cell sequencing has been rapidly developing in recent years and provides cancer research with unparalleled opportunities to improve cancer care. A variety of novel technologies and data analysis methods are available to discern underlying molecular mechanisms in cancer; however, these rapidly developing multi-omics analyses are still at an early stage [1]. There are many examples of individual and multi-omics profiling of single cells with various advantages and disadvantages, and there is also a need to integrate temporal and spatial information for individual cells [2].

Integration is indeed a challenge and this also includes creating large databases that contain annotated single-cell sequencing data for various tumor and normal cells, including humans and other species, from various projects. Large amounts of next-generation sequencing data residing in ‘silos’ has been a challenge [3] and single-cell sequencing will also need to be considered when creating an integrated resource to support precision medicine. Multiple single-cell genomics consortia, for example, the Human Cell Atlas (HCA), have been created to integrate, harmonize, and share single-cell data [4]. It will be important to consider how all these resources may be used together.

The ultimate goal is to improve cancer care. Single-cell sequencing methods should have clinical implementations in the future for personalized medicine, for cancer as well as other diseases [5]. Data analytics will be key and how quickly and efficiently these technologies become available for personalized medicine may depend on how effectively we solve issues related to data integration and analytics. Integrated analytics will also aid discovery of new targets and development of synergistic combination therapies, including enhanced immunotherapy strategies [6]. Moving this field forward will require solutions for additional challenges [7]. Our aim is that this Special Issue will help promote the discussion on topics central in this area through publishing novel tools, methods, resources, insights, and applications.
